# Do Therapists Google Their Patients? A Survey Among Psychotherapists

**DOI:** 10.2196/jmir.4306

**Published:** 2016-01-05

**Authors:** Christiane Eichenberg, Philipp Y Herzberg

**Affiliations:** ^1^ Department of Psychology Sigmund Freud University Vienna Austria; ^2^ Professorship of Personality Psychology and Psychological Diagnostics University of the Federal Armed Forces Hamburg Hamburg Germany

**Keywords:** patient-targeted googling (PTG), Internet, patient-therapist relationship, professional-patient relationship, professional guidelines, educational curriculum

## Abstract

**Background:**

The increasing use of the Internet and its array of social networks brings new ways for psychotherapists to find out information about their patients, often referred to as patient-targeted googling (PTG). However, this topic has been subject to little empirical research; there has been hardly any attention given to it in Germany and the rest of Europe and it has not been included in ethical guidelines for psychotherapy despite the complex ethical issues it raises.

**Objective:**

This study explored German psychotherapists’ behavior and experiences related to PTG, investigated how these vary with sociodemographic factors and therapeutic background, and explored the circumstances in which psychotherapists considered PTG to be appropriate or not.

**Methods:**

A total of 207 psychotherapists responded to a newly developed questionnaire that assessed their experience of and views on PTG. The study sample was a nonrepresentative convenience sample recruited online via several German-speaking professional therapy platforms.

**Results:**

Most therapists (84.5%, 174/207) stated that they had not actively considered the topic of PTG. However, 39.6% (82/207) said that they had already looked for patient information online (eg, when they suspected a patient may have been lying) and 39.3% (81/207) knew colleagues or supervisors who had done so. Only 2.4% (5/207) of therapists had come across PTG during their education and training.

**Conclusions:**

It is essential to provide PTG as a part of therapists’ education and training. Furthermore, the complex problems concerning PTG should be introduced into codes of ethics to provide explicit guidance for psychotherapists in practice. This report provides initial suggestions to open up debate on this topic.

## Introduction

The Internet has become an essential and frequently used medium for retrieving diverse information about people and organizations. There may be a variety of reasons for this, including private curiosity (some people may wish to look up a friend from school to find out what she is doing now) or there may be job-related reasons for exploring the Web. But what about professional relationships between therapists and patients? Therapists use the Internet for assistance in everyday work tasks; for example, most psychotherapists use email as well as mobile communication to contact their patients as described by Eichenberg and Kienzle [1]. Furthermore, Zur et al [2] have reported an increasing prevalence of “deliberate self-disclosure” by therapists who maintain a presence on the Internet. For patients, this can make it easier to choose a therapist as Eichenberg et al [3] found in a national survey in Germany: nearly two-thirds (63.5%) of Internet users search the Web for health-related information and 43.7% could imagine seeking help online in cases of emotional distress (eg, obtaining the contact data of a resident psychotherapist).

Could such information interfere with the relationship between therapist and patient? Facebook, Xing, Twitter, etc, open up further possibilities for information use and thereby possibilities for abuse. The Internet represents countless ways of using information without the slightest moral or ethical consideration of any effects this might have on therapeutic relationships. According to Ensher et al [4], just as managers look for background information on the Internet about their potential employees, psychotherapists also look up their potential patients as assumed, but not empirically proven, by Clinton et al [5]. An empirical study revealed that a large majority of psychology graduates (81%) report using online social networks, although approximately 27% of psychotherapists and therapists in education look for online information on their patients [6]. This phenomenon is described by Clinton et al [5] as “patient-targeted googling” (PTG). However, neither the American guidelines for psychotherapists (American Psychological Association [APA]) [7] nor the German guidelines (Berufsverband Deutscher Psychologen [BDP]) [8] give any explicit guidance on this issue.

The APA recommends that psychologists should “...respect the dignity and worth of all people, and the rights of individuals to privacy, confidentiality, and self-determination” (p 4 [7]). This includes keeping intrusions on privacy by the therapist to a minimum. Comparing the US and German guidelines does not lead to any further or more explicit conclusion: the ethical guidelines for psychotherapists in Germany do not explicitly mention the phenomenon of online research of patient information. But its indirect implementation can be found in the professional code of the trade union of German psychologists [8]. It states that psychologists are only allowed to collect, save, and use client or patient data that is in line with the treatment order. The necessity for a bond of trust and the idea that psychologists should inform their patients about all substantial procedures at all steps in the treatment and ask for patients’ agreement are well established [8]. But what would be considered a “substantial procedure”? When can the bond of trust be interrupted? When could a therapist satisfy their curiosity instead of following psychotherapeutic orders?

To answer these questions, it seems reasonable to examine PTG. As yet, there have been only 2 articles that have presented empirical material on this [5,6], so more empirical data are needed. Therefore, the aim of this study was to investigate PTG in Germany from the perspective of therapists. Specifically, we intended to answer the following questions:

1. What reasons correspond with what type of information?
2. How much information about PTG do psychotherapists receive in their education or through professional experience?
3. When do psychotherapists consider PTG to be appropriate and inappropriate?
4. What reasons are there for and against PTG in general?
5. Do attitudes differ according to sociodemographic factors?
6. In what ways is the information gained online used for therapeutic reasons?
7. To what extent do therapists know about patients who search for online information about them?
8. Do therapists take precautions to control the information about them available on the Internet?

## Methods

### Recruitment

The study sample was a nonrepresentative convenience sample recruited online via several professional therapy platforms. (For a discussion of the scientifically proven quality of Web-based studies, see Gosling et al [9].)

### Questionnaire

A questionnaire was administered online, generated using the online tool Unipark [10]. It included 36 items organized into 3 sections: (1) sociodemographic data, including age, sex, professional experience, type of psychotherapeutic education, and frequency of Internet use; (2) experience of and attitudes toward PTG structured into open and closed questions with a focus on knowledge of the PTG phenomenon followed by 7 further items to be answered by the participants who had experience with PTG; and (3) online research by patients, including the reactions and experiences of therapists who reported patients searching for information about them via the Internet.

### Statistical Analysis

In addition to descriptive statistical methods, inference statistical methods were used for the closed questions (correlation analysis and chi-square test); content analysis was used to analyze the open answers for some of the questions [11]. Inductive categories were designed for single questions, whereas coding entities were defined by its oneness of sense. The data were analyzed with SPSS version 19 and PASW Statistics version 18.

### Sample

The sample included 207 psychotherapists (15/207, 7.2% in education), of whom 67.6% (140/207) were female, a similar proportion to the distribution of medical and psychological therapists in Germany [12]. The mean age of participants was 45.00 (SD 10.49) years, which is younger than the mean age of German therapists (mean 53 years). This may be a result of the Web-based study conception, given that younger therapists may be more inclined to use the Internet. More than half (51.2%, 106/207) of the therapists were licensed by health insurance and, on average, they had spent longer than 12 years in professional life. More than two-thirds (70.1%, 145/207) of the therapists worked in their own practice or a group practice with the rest located in clinics or other facilities. The majority of respondents (70.1%, 145/207) worked with adults in a single therapeutic setting; the types of therapy practiced included (multiple answers were allowed) cognitive behavioral therapy (51.2%, 106/207), psychodynamic psychotherapy (44.0%, 91/207), psychoanalysis (25.1%, 52/207), systemic psychotherapy (12.1%, 25/207), and various types of education (18.8%, 39/207). Most therapists (85.0%, 176/207) said that they used the Internet at least once a day for professional reasons; therefore, high Internet affinity can be assumed. In a professional setting, 96.1% (199/207) used the Internet to access factual information and 82.1% (170/207) to exchange information with colleagues.

## Results

### To What Extent Did the Psychotherapists Carry Out Patient-Targeted Googling?

Most therapists (84.5%, 174/207) stated that they had not actively considered the topic of PTG. Only 2.4% (5/207) had heard about PTG as part of their education or advanced training. Nevertheless, 39.6% (82/207) said that they had already looked for patient information online and 39.3% (81/207) knew colleagues or supervisors who had done likewise. Of the remaining 60.5% (125/207) who claimed that they had not looked for patient information online, 90.4% (113/125) would not do so even if regulations and the law were clarified. The main reasons for this were ethical doubts (36.9%, 42/113) and lack of confidence in the Internet as a source of reliable information (32.8%, 37/113). At the same time, a quarter (24.8%, 28/113) did not even want to know about the online information and another quarter (23.0%, 26/113) claimed that they were not willing to do the extra work involved. Only 13.3% (15/113) supposed that patients would not agree with such behavior. (Respondents were allowed to give multiple answers.)

### Did the Psychotherapists With Experience of Patient-Targeted Googling Differ From the Others in Sociodemographic Factors? What Did Patient-Targeted Googling Involve? What Kinds of Information Were Obtained?

Data analysis showed no effect of sex or age on PTG. Nor were the type of therapeutic treatment provided or the age class of patients linked to online information research. Only the frequency of Internet use was significantly correlated with the probability of PTG (*r*=.18, *P*<.001).

The more than one-third of therapists (39.6%, 82/207) who looked online for patient information did so for a mean 5.8 cases (SD 8.8). Three-quarters (76%, 62/82) did this without the patients’ permission, whereas 21% (17/82) gained permission from their patients often or all the time to search for the information online. Only 4% (3/82) stated that they only looked for the information together with their patients. Home pages, blogs, and social networks were of greatest interest for obtaining information.

### How Much Information About Patient-Targeted Googling Did Psychotherapists Receive in Their Education or Professional Experience?

Only 3 of 207 therapists (1.4%) received information about PTG during their education. They came from different therapeutic backgrounds, so no conclusions could be drawn from this. Only 2 of 207 therapists (1.0%) heard about PTG during advanced training. Overall, only 15.5% (32/207) of therapists had consciously considered the topic of PTG.

### When Did Psychotherapists Consider Patient-Targeted Googling to Be Appropriate and Inappropriate? What Reasons Were There For and Against Patient-Targeted Googling in General? Did Attitudes Differ According to Sociodemographic Factors?

Irrespective of whether they themselves had carried out PTG, the therapists were asked about their attitude toward it. More than one-third (38.6%, 80/207) of therapists thought that searching the Web for patient information was unimaginable; for them, there was no possibility of them doing so. Approximately two-thirds felt differently and agreed that certain situations could indicate or allow PTG: 18.1% (38/127) of therapists would agree with using PTG in consensual agreement with the patient, 13.4% (28/127) would allow PTG in circumstances where there was imminent danger, 9.5% (12/127) would agree if there were a reasonable suspicion that the patient was lying, and 8.7% (11/127) allowed for the possibility of PTG at all times (multiple responses were allowed in the questionnaire).

The analysis of the open questions (Textbox 1) illustrated the special justifications for situations when therapists considered PTG to be appropriate. One therapist, for example, tried to gain information from the Internet about a suicidal patient who wanted to kill himself with a gun. To avoid danger, the therapist looked for membership at a shooting or gun club to check whether the patient had access to any kind of firearm and the knowledge of how to use it. Other situations considered legitimate by therapists included finding missing contact details or looking up information with the agreement of the patient.

**When do psychotherapists consider PTG to be appropriate? Response categories and sample quotes to open questions (n=53 therapists with n=59 answers).**

1. Danger to self and others (n=22)

- “Acute danger to self and others.”
- “If a patient tries to endanger others (run amok etc).”
- “Sexual offenders with treatment orders.”
- “When I myself as a therapist am clearly threatened.”
- “Planned suicides.”

2. If discussed/desired (n=9)

- “If my patient desires that I look at his webpage.”
- “At the patient’s request.”
- “If the patients’ request is comprehensible for me.”

3. Missing extra information (n=8)

- “To get more information.”
- “To complete anamnesis.”
- “For biographical info.”

4. Formalities (n=7)

- “Unpaid bills”
- “If I only had the patient’s old phone number and I need the new one.”
- “Checking an address or phone number.”

5. Patients in public life (n=5)

- “Patient is part of public life and newspaper articles (defamations) are a subject of therapy.”
- “Patients who have a public life and assume that you are preinformed when you aren’t (eg, you are not informed about footballers if you are not a football fan yourself).”

6. Content of therapy (n=5)

- “Interest in how patients present themselves online.”
- “Suspicion of cybermobbing.”
- “Young people use the Internet differently to us elder people (often uncritical and uncensored).”

7. Other (n=3)

- “After finishing the therapy I would be all right with it.”
- “Pure curiosity.”

There were relationships between the therapists’ approaches to therapy and their evaluation of PTG’s legitimacy. Therapists trained in psychodynamic-oriented therapy (χ^2^_1_=15.5, *P*<.001) or psychoanalytical therapy (χ^2^_1_=17.8, *P*<.001) responded significantly more often than cognitive behavioral therapists (χ^2^_1_=13.4, *P*<.001) that PTG is inappropriate in all situations. These differences may originate in specific aspects of asymmetric therapist-patient relationships in psychodynamic approaches to psychotherapeutic treatment. Analytic reasons (eg, the rule of abstinence) may not only have an impact on ethics, but also on general techniques in treatment. For other criteria, no associations were found.

Analysis of the therapists’ self-written answers on reasons that justify PTG (Textbox 2) showed that the most commonly mentioned reasons in favor of PTG were for a change of perspective, which should lead to a better understanding of the patient and recognition that Internet-based information was freely available. Verification of data and checking for suspected lies were also given as reasons to search for patient information, as was curiosity.

**Justifications for researching a patient’s information via the Internet. Response categories and sample quotes to open question (n=132 therapists with n=149 answers).**

1. Better understanding because of more information and a change of perspective (n=34)

- “Better understanding of the patient’s social environment.”
- “External, more widespread information that is not controlled directly by the patient.”
- “eg, Patient is a refugee and I can imagine better his home and be more empathic.”

2. Therapy-relevant information is on the Internet (n=17)

- “To be authentic to patients who attach importance to their Web presence.”
- “How do patients present themselves on the Web?”
- “To gather information about how patients present themselves or so that they don’t overlook that their self-expression can be seen by others.”

3. Online information is public (n=8)

- “Anyone who provides their personal data on the Internet implicitly gives permission for this to be seen by others. That’s why I don’t need to ask for the patient’s permission.”
- “It is about information which belongs to patients, normally provided by them; and if not it is still part of patients’ expression of personality.”
- “Anyone who provides online information needs to expect that it will be read.”

4. On the request of the patient (n=8)

- “Permission of patient after agreement or request.”
- “After the patient’s explicit request.”

5. Curiosity (n=7)

- “If it is an interesting patient and you want to get to know more about him.”
- “Sometimes, once in a blue moon, I do it out of curiosity. But I don’t think it is essential or reasonable. In the end it is only one option of investigation: I want to gather information about a patient, eg by doing a third-party review of the patient’s case history without consent.”

6. Controlling patients’ statements (n=6)

- “A kind of reality check. Is the patient really as famous as he says?”
- “Verifying patients’ information about their activities and occupation. I have only done that in the case of narcissistic male patients and got a feeling of greater objectivity later on.”

7. Suspicion of lying and concealment (n=5)

- “Suppressing facts such as criminal proceedings.”
- “Trying to gain secretive factual information, to clear up discrepancies.”

8. Nothing (n=64)

- “Currently I cannot imagine any situation where Internet research could be helpful for the therapeutic process.”
- “Under the aspect of a relation of trust: nothing.”
- “I do not know any reason!”

In their arguments against PTG (Textbox 3), therapists stated that the relationship of trust could be damaged and that patients should also have the right to decide for themselves what information they wanted to share. Protection of privacy and doubts about the real advantage and usefulness of the information were mentioned by many of the therapists who were against PTG. The risk to countertransference in therapeutic work was also an issue raised.

**Arguments against researching patients’ information on the Internet. Response categories and sample quotes to open question (n=103 therapists with n=128 answers).**

1. Disturbance in the trust relationship (n=39)

- “The open relationship of trust with patients. If relatives of patient provide written or oral information, for example, I would handle it the same open way as if it was obtained from Internet research.”
- “It harms the bond of trust; patients don’t “lie” to me—they deceive themselves.”

2. Patients’ self-determined information control (n=20)

- “Patients need to be in control of what they say.”
- “Personal rights, privacy.”
- “The right to lie.”

3. Border violation/ensuring privacy (n=17)

- “Ensuring privacy.”
- “The right of patients to ‘privacy’—to appear in therapy the way they want to and need to.”

4. Rule of abstinence and curiosity (n=13)

- “The rule of abstinence for psychotherapists as example.”
- “Personal curiosity.”

5. Manipulation/lack of impartiality (n=12)

- “My principle: all I learn about my patients is what I am told by them, not information obtained behind their back. This influences the unconscious therapeutic relationship.”
- “Corruption of therapeutic neutrality in front of patients by having information they might not have wanted to give to me—concealed information could have a special function.”

6. Doubtful reliability or usefulness of the information (n=11)

- “Not objective, only parts of the whole, not possible to demonstrate validity.”
- “Lots of trash on the Internet”
- “You don’t get the information you really needed for therapy.”

7. Acting of countertransference (n=7)

- “Substantial disturbance in the relationship of trust as well as in transference and countertransference.”
- “I consider the research of such data to be a professional and ethical problem. Professional, because instead of analyzing the countertransference you start acting; and ethical because of violation of abstinence and destruction of the relationship of trust/protected area.”

8. Nothing (n=9)

- “Precisely nothing.”

The summarized answers to the open questions show an ambivalent attitude toward PTG in the therapists’ behavior and thought. Ignoring the answers in the questionnaire that were neither for nor against PTG, there were 85 responses in total justifying PTG and 119 arguments against it.

A similar result was also found with the answers to closed questions (Table 1). Of the arguments to justify PTG, the 2 that received the most agreement were that therapists should have access to freely available Internet information and that therapists should be in a position to separate their curiosity from necessity. More support can be found for the arguments against PTG, with most therapists agreeing that there were risks of curiosity being the motivation and there being the potential of harming the relationship of trust with the patient and of acting out countertransference. Table 1 sheds more light on therapists’ ambivalent opinions.

**Table 1:** The proportion and number of therapists who agreed or disagreed with patient-targeted googling (multiple responses were allowed; N=207).

Specific statements about PTG		n (%)
**Justifying PTG**		
	Information is provided online for all people. Online information is information shared with the therapist as well.	58 (28.0)
	A good therapist can differentiate between curiosity and therapeutic need and does not run the risk of doing PTG with intrinsic motivation.	39 (18.8)
	The Internet is in cases of emergency the quickest available resource to use.	32 (15.5)
	Decisions have to be made on the basis of patient benefit. This even includes seeking additional information a patient does not want or is unable to give but which might accelerate help.	24 (11.6)
	None of the above (positive) statements.	77 (37.2)
**Against PTG**		
	Personal curiosity is certainly a motivation for PTG (perhaps unconsciously).	125 (60.4)
	The bond of trust between patient and therapist collapses because of PTG.	97 (46.9)
	Internet information is not reliable in the case of patient’s inquiry.	80 (38.6)
	Therapists are not allowed to gain information they were not officially provided with; this includes information from the Internet.	65 (31.4)
	None of the above (negative) statements.	21 (10.1)

### How Was the Online Information Used Therapeutically?

Nearly two-thirds (65%, 53/82) of therapists who used PTG did not annotate their findings in the patient’s record because they considered the information to lack importance or saw it as mirroring information that was already known. The rest of the therapists referred to fulfilling obligatory documentation requirements (10%, 8/82) or to sporadic documentation (26%, 21/82). Patients were not consulted about online research in 38% (31/82) of cases for these reasons. Some emotional reasons of therapists also came across; for example, the pursuit of patients or curiosity should not be part of the therapy. Online results were discussed within therapy sessions (always: 31%, 25/82; sometimes: 32%, 26/82) to clarify mismatches mostly in cases where the online information was relevant to the therapy.

In the opinion of nearly one-third (32%, 26/82) of the therapists, no important or interesting details about their patients were found on the Web. PTG is seen by many as having a potential therapeutic use in allowing a better understanding to be gained of the public roles of some patients as well as providing an interesting focus on patients’ self-expression. Furthermore, it has been understood as providing “certification for issues discussed in therapy” to make sure the patient is being understood in the correct way. A few therapists who did PTG said that personal interest certainly provided high motivation for online investigation.

### To What Extent Do Therapists Know About Patients Who Search for Online Information About Them?

Therapists raised more concerns about being “googled” than they did about PTG; 91.3% (189/207) said that they had already thought about this issue. In the answers given to open questions, views were expressed that patients’ curiosity is justified, whereas the negative perceptions about being googled focused on the violation of privacy, concerns about the control of information provided online, and worries about potential negative rating of therapists online. Of the therapists who had never considered the topic before (8.7%, 18/207), some said that they had no online information so the topic did not concern them. More than half of the therapists (54.6%, 113/207) were researched online by a patient at least once or were content with patients researching them. There was a significant difference between the sexes; male therapists were more often the subject of research than women (χ^2^_1_=6.8, *P*=.009) and correlation analysis showed that the frequency of being researched increased with the therapists’ length of time in therapeutic practice (*r*=.31, *P*<.001). There was also a significant correlation between the amount of time therapists spent using the Internet and the number of times they were targeted for online research by patients (*r*=.22, *P*=.002). Results also suggest that a patient’s interest in knowing his or her therapist seems to increase with treatment in long-term therapy.

### Did the Therapists Take Precautions to Control the Information Available About Them on the Internet?

Most therapists (58.9%, 122/207) controlled the information available about them on the Internet for security reasons by uploading only carefully selected information. Almost one-third (29.5%, 61/207) preferred not to post any personal information online and 46.4% (96/207) did use search engines to check for the online information available about them. Nearly 10% (21/207) did not think it was necessary to protect themselves or had never thought about this. Only 4.3% (9/207) used Google alerts (a tool for online Web monitoring of new content that can also be used for names, etc) for searching their own name (to see when any new entries became available online), whereas 7.7% (16/207) employed other methods to keep updated. Only 40.5% (84/207) declared their membership to social networks and only 9 of 207 (4.3%) therapists allowed unrestricted access to their social network accounts, with most members of social networks sharing their details only with friends or using false names or nicknames.

## Discussion

### Principal Results

This study investigated PTG by German psychotherapists, focusing on the experience of and attitudes toward PTG for a sample of therapists. All health care professionals have the option of using the Internet for looking up information about their patients, but this has particular relevance for psychotherapeutic relationships. Not only must psychotherapists keep in mind the ethical aspects of PTG (which would be of concern to all health care professionals), but they must also consider their therapeutic relationship with their patients, which raises many more aspects of concern. For example, what might be the effects of PTG on the therapeutic relationship, such as in countertransference that is not reflected but acted out? For certain, PTG influences the therapeutic relationship on a very particular individual basis, and often with profound consequences, such as harm to the bond of trust. When could PTG be seen as a symptom of a failed therapeutic relationship? Can PTG be understood as a violation of borders when it comes to the terms of the rule of abstinence on the basis of the code of ethics of psychological psychotherapists in Germany? Stellpflug and Berns [13] state that the relationship of trust between therapist and patient should not be abused for the satisfaction of the therapist’s own interests and needs; this would mean that there has been a clear violation of guidelines when, for instance, a therapist searches on the Internet for information on their patients out of curiosity. Conversely, are there any reasons or situations that legitimate PTG? If its use is legitimate, how should the therapist proceed with information found on the Internet?

Results of the current study show that the majority (84.5%) of therapists who responded had not actively engaged with the topic of PTG. Yet 39.6% said that they had already searched for patients’ online information, which proves that there has been use of the Internet as a source of information about patients without full consideration. The correlations found between research activities and the general use of Internet are not surprising: frequent use of the Internet and the consequent integration of this medium into daily life make its use in other contexts more likely. In contrast, the relationship found between the psychotherapy orientation of the therapist and attitudes toward PTG is more notable: psychodynamic-oriented therapists were much more often of the opinion that there were no justified reasons for PTG than were their behaviorally trained colleagues. Given that PTG is almost never discussed during their therapeutic education, these differences in attitude must be due to broader aspects of their therapeutic positioning, such as their conception of the working alliance, the therapeutic relationship, the rule of abstinence, or privacy. Further studies regarding this are needed. In addition to studies that focus on the psychotherapist taking into account the bidirectional bond between psychotherapist and the patient, there is also a need to focus on the patient’s perspective: the possibility of patients using the Internet to gain information about psychotherapists and how to respond to this. As well as describing the use of information gained from the Internet about therapists and patients, there would be value in discussing the clinical utility of this information-seeking behavior.

### Limitations

In general, PTG is seldom discussed and has not been the object of empirical scientific analysis. Therefore, this survey should only be thought of as a first explorative study to improve understanding of PTG. Due to the data collection procedure used, this study did not involve a representative sample of Internet-using psychotherapists in Germany (let alone worldwide). We were not able to test whether PTG was over- or underestimated in our sample. However, methodical studies have shown that Web-based surveys can achieve comparable response rates to questionnaires delivered by mail [14]. A theoretical bias also cannot be excluded; therapists who are interested in ethics may be overrepresented in the study and their interest in ethics may be driven by the idea of a therapeutic use of the Internet. However, there is no evidence of any self-selection of participants of this kind. A further limitation was that the therapist-patient dyad was not investigated. This will clearly need to be looked at in future studies.

### Implications

In future, discussion of PTG should become part of therapists’ education and training. For instance, as well as giving information about the prevalence and circumstances of PTG gained from empirical studies such as this one, emerging therapists should be pointed toward the influence of PTG on the therapist-patient relationship. This could be accomplished through a discussion of the pros and cons of PTG, and augmented by case studies and analyses of the feelings of countertransference and transference of self. Furthermore, the complex nature of problems related to PTG should be introduced into codes of ethics to provide explicit guidance for therapists in practice. In the first therapy session, the role of modern media in the therapeutic process should be discussed (eg, whether the therapist can be contacted via email or text messaging or whether mental health programs should be a part of therapy); in this context, the need to search online for information about each other can be adressed. Potential implicit expectations of patients (eg, in searching for the therapist on Facebook and requesting to be accepted as a friend) open up new and wide fields that need to be understood to maintain quality in patient treatment.

## Abbreviations

PTG: patient-targeted googling
